# Complete mitochondrial genome of *Pseudocrangonyx joolaei* (Crustacea: Amphipoda: Pseudocrangonyctidae)

**DOI:** 10.1080/23802359.2020.1797592

**Published:** 2020-07-25

**Authors:** Chi-Woo Lee, Takafumi Nakano, Ko Tomikawa, Gi-Sik Min

**Affiliations:** a Department of Biological Sciences, Inha University, Incheon, Korea; b Department of Zoology, Graduate School of Science, Kyoto University, Kyoto, Japan; c Department of Science Education, Graduate School of Education, Hiroshima University, Higashihiroshima, Japan

**Keywords:** Amphipoda, cave, complete mitogenome, Korea, *Pseudocrangonyx joolaei*

## Abstract

The complete mitochondrial genome of a subterranean pseudocrangonyctid amphipod, *Pseudocrangonyx joolaei*, was determined in this paper. The complete mitogenome of *P*. *joolaei* was 14,814 bp in length with the typical 13 protein-coding genes, 22 transfer RNAs, two ribosomal RNAs, and a control region (CR). The gene order of *P*. *joolaei* was unique in that the CR was an inversion, and the gene order of *Pseudocrangonyx* was not concordant when compared to that of *P*. *daejeonensis*, a subterranean amphipod found in Korea. A maximum-likelihood tree, constructed based on 26 eumalacostracan mitogenomes, confirmed that *P*. *joolaei* supported monophyly in the family Pseudocrangonyctidae and is most closely related to the superfamily Crangonyctoidea.

Amphipods are the most diverse group in groundwater communities, and the restricted habitat and distribution of stygobitic amphipods are important for the study of biogeography (Holsinger 1994). The genus *Pseudocrangonyx*, belonging to the superfamily Crangonyctoidea, is an important component of Holarctic subterranean habitats (Holsinger 1993, 1994), and is the most diverse taxon among the subterranean-amphipod genera found in East Asia, i.e., the Korean Peninsula, Japan, Eastern China, and the Russian Far East (Sidorov and Holsinger 2007; Tomikawa and Nakano 2018). However, the evolutionary history of *Pseudocrangonyx* is poorly studied, with most studies focusing on species revision and discovery (Zhao and Hou 2017). To date, the *Pseudocrangonyx* mitogenome has been fully determined only in *P*. *daejeonensis*, and the gene arrangement of *P*. *daejeonensis* shows many differences with respect to the typical pan-crustacean ground pattern (Lee et al. 2018). Moreover, the gene arrangement in the newly determined *P*. *joolaei* was inconsistent from that of *P*. *daejeonensis* despite both belonging to the same genus. Previous studies on subterranean amphipods inhabiting Europe and North America have highlighted the variability of the gene order in their mitogenome (Bauzà-Ribot et al. 2009; Aunins et al. 2016). Therefore, it remains unknown whether the mitogenome order among *Pseudocrangonyx* amphipods is variable. Accordingly, additional knowledge regarding the mitogenome of another *Pseudocrangonyx* species will lead us to a better understanding of the evolutionary history of gene arrangement in stygobitic amphipods.

Individuals of *Pseudocrangonyx* were collected from cave groundwater in Korea (36°47.13′N, 127°57.76′E). Mitochondrial DNA extraction, sequencing, and gene annotation were performed using the methods described by Song et al. (2016). The extracted mitochondrial DNA has been deposited in the DNA collection at the National Institute of Biological Resources, Incheon, South Korea (deposit no. NIBRGR0000619445). A maximum-likelihood tree was constructed using IQ-tree 1.6.3 with mtZOA + F+R6 model (Nguyen et al. 2015; Chernomor et al. 2016), based on the concatenated sequences of 10 protein-coding genes (*ATP6, COX1, COX2, COX3, CYTB, NAD1, NAD2, NAD3, NAD4*, and *NAD5*) from 26 eumalacostracan species, including the present sequence and two isopods as outgroup taxa. The *cox1* sequence of the extracted DNA was concordant with that from the paratype of *P*. *joolaei* (NCBI accession no. LC467002; Lee et al. 2020), and thus, the taxonomic identity of the present material was unquestionably clarified.

The complete mitogenome of *P. joolaei* (NCBI accession no. MT211951) was 14,814 bp in length and contained 13 protein-coding genes, 22 transfer RNAs, two ribosomal RNAs, and a control region. The gene arrangement of the complete mitogenome of *P*. *joolaei* was almost identical to the typical pan-crustacean ground pattern except the location of a control region. The control region of *P*. *joolaei* was found to be located between *NAD1* and *16S rRNA*. According to the gene arrangements of the amphipod revealed so far, the translocation of the control region is found only in *Pseudocrangonyx*; the exception being two lysianassoidean species inhabiting the deep sea, *Eurythenes magellanicus* and *E*. *maldoror* (Li et al. 2020).

The obtained maximum-likelihood tree showed monophyly of the family Pseudocrangonyctidae and this lineage was most closely related to a clade consisting of *Stygobromus* amphipods belonging to the superfamily Crangonyctoidea (Figure 1).

**Figure 1.**
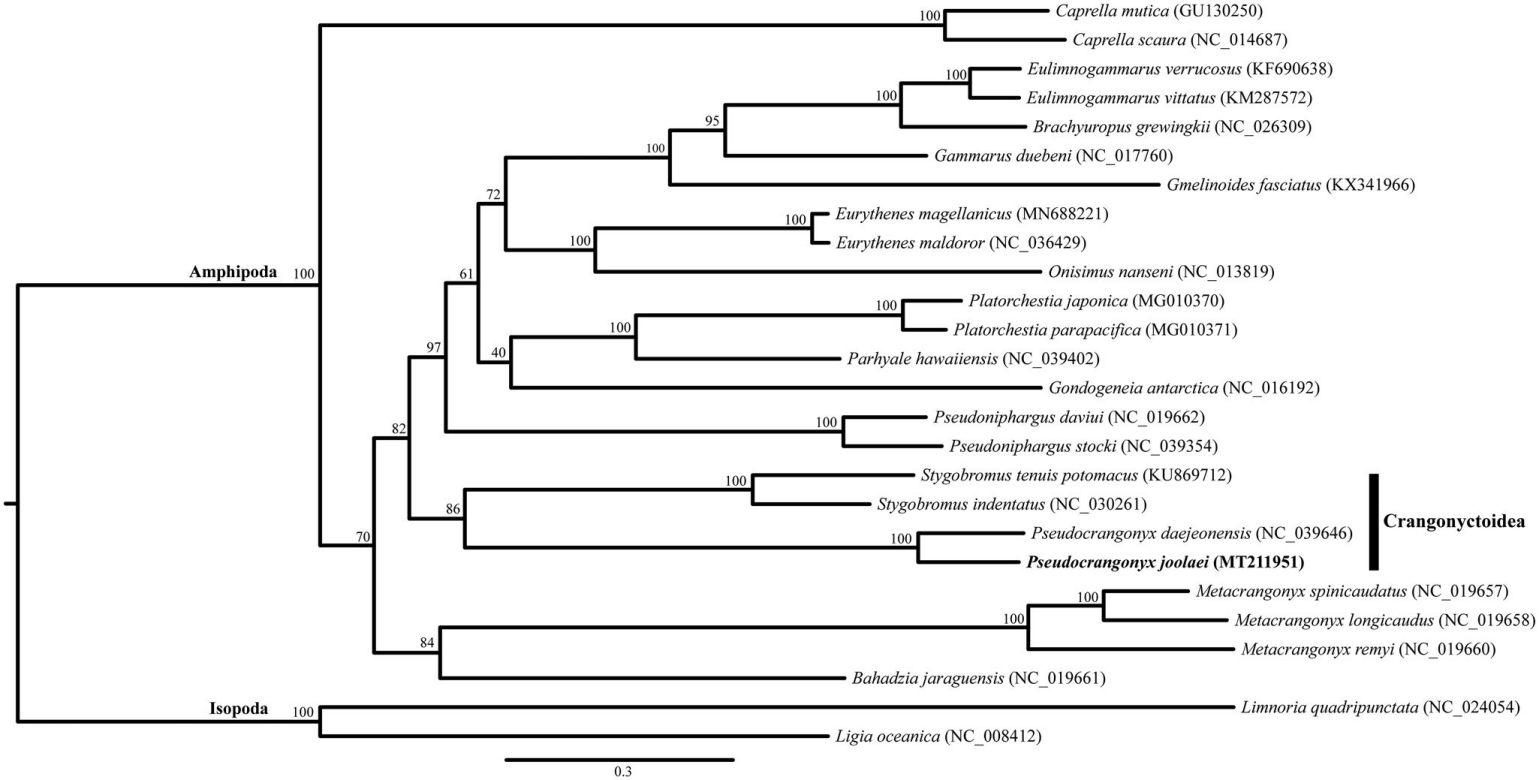
Maximum-likelihood (ML) tree based on the mitogenome sequence of *Pseudocrangonyx joolaei* (MT211951) with 25 other eumalacostracan species. The bootstrap supports are shown on each node.

## Data Availability

The data that support the findings of this study are openly available in GenBank at https://www.ncbi.nlm.nih.gov, reference number MT211951.

## References

[CIT0001] Aunins AW, Nelms DL, Hobson CS, King TL. 2016. Comparative mitogenomic analyses of three North American stygobiont amphipods of the genus *Stygobromus* (Crustacea: Amphipoda). Mitochondrial DNA Part B. 1(1):560–563. 33473556 10.1080/23802359.2016.1174086 PMC7800481

[CIT0002] Bauzà-Ribot MM, Jaume D, Juan C, Pons J. 2009. The complete mitochondrial genome of the subterranean crustacean *Metacrangonyx longipes* (Amphipoda): a unique gene order and extremely short control region. Mitochondrial DNA. 20(4):88–99. 19513930 10.1080/19401730902964417

[CIT0003] Chernomor O, von Haeseler A, Minh BQ. 2016. Terrace aware data structure for phylogenomic inference from supermatrices. Syst Biol. 65(6):997–1008. 27121966 10.1093/sysbio/syw037 PMC5066062

[CIT0004] Holsinger JR. 1993. Biodiversity of subterranean amphipod crustaceans: global patterns and zoogeographic implications. J Nat Hist. 27(4):821–835.

[CIT0005] Holsinger JR. 1994. Pattern and process in the biogeography of subterranean amphipods. Hydrobiologia. 287(1):131–145.

[CIT0006] Lee C-W, Nakano T, Tomikawa K, Min G-S. 2018. The complete mitochondrial genome of *Pseudocrangonyx daejeonensis* (Crustacea: Amphipoda: Pseudocrangonyctidae). Mitochondrial DNA Part B. 3(2):823–824. 33474336 10.1080/23802359.2018.1495116 PMC7799821

[CIT0007] Lee C-W, Tomikawa K, Nakano T, Min G-S. 2020. A new species of the genus *Pseudocrangonyx* (Crustacea: Amphipoda: Pseudocrangonyctidae) from Simbok Cave. Korea. Zootaxa. 4731(3):zootaxa.4731.3.2–334. 32230294 10.11646/zootaxa.4731.3.2

[CIT0008] Li JY, Liao YW, Li J, He LS. 2020. The complete mitochondrial genome of the deep-sea amphipod *Eurythenes magellanicus* (Crustacea: Amphipoda: Lysianassidae). Mitochondrial DNA Part B. 5(1):337–339. 10.1080/23802359.2019.1703573 PMC7748488 33366546

[CIT0009] Nguyen L-T, Schmidt HA, von Haeseler A, Minh BQ. 2015. IQ-TREE: a fast and effective stochastic algorithm for estimating maximum-likelihood phylogenies. Mol Biol Evol. 32(1):268–274. 25371430 10.1093/molbev/msu300 PMC4271533

[CIT0010] Sidorov D, Holsinger JR. 2007. *Procrangonyx stygoedincus*, a new species of subterranean amphipod (Pseudocrangonyctidae) from the Far East of Russia, with remarks on biogeographic relationships. Crustac. 80(4):417–430.

[CIT0011] Song J-H, Kim S, Shin S, Min G-S. 2016. The complete mitochondrial genome of the mysid shrimp, *Neomysis japonica* (Crustacea, Malacostraca, Mysida). Mitochondrial DNA A DNA Mapp Seq Anal. 27(4):2781–2782. 26114317 10.3109/19401736.2015.1053064

[CIT0012] Tomikawa K, Nakano T. 2018. Two new subterranean species of *Pseudocrangonyx* Akatsuka & Komai, 1922 (Amphipoda: Crangonyctoidea: Pseudocrangonyctidae), with an insight into groundwater faunal relationships in western Japan. J Crustacean Biol. 38(4):460–474.

[CIT0013] Zhao S, Hou Z. 2017. A new subterranean species of *Pseudocrangonyx* from China with an identification key to all species of the genus (Crustacea, Amphipoda, Pseudocrangonyctidae). ZooKeys. 647:1–22. 10.3897/zookeys.647.11192 PMC5345347 28325961

